# Effectiveness of immunosuppressant use for the treatment of immune checkpoint inhibitor-induced liver injury: A systematic review and meta-analysis

**DOI:** 10.3389/fonc.2023.1088741

**Published:** 2023-03-24

**Authors:** Kefan Chen, Junhao He, Jing Xu, Jie Chen

**Affiliations:** ^1^ Department of Pharmacy, The First Affiliated Hospital, Sun Yat-sen University, Guangzhou, China; ^2^ School of Pharmaceutical Sciences, Sun Yat-sen University, Guangzhou, China; ^3^ Department of Pharmacy, Dermatology Hospital, Southern Medical University, Guangzhou, China

**Keywords:** immunosuppressant, immune-mediated liver injury caused by checkpoint inhibitors, treatment management, corticosteroids, response rate

## Abstract

**Background:**

Immune-mediated liver injury caused by checkpoint inhibitors (ILICI) is a challenging clinical management issue. Although immunosuppressants are widely used to manage ILICI, no large-scale studies have proved definitive evidence for the most effective form of patient management.

**Aim:**

Analysis of the effectiveness of immunosuppression for immune-related liver injury.

**Methods:**

We performed a systematic review and meta-analysis of the clinical outcomes of immunosuppressive treatment of ILICI patients. A literature search of PubMed, Ovid, and Cochrane Library was completed for dates from 2000 to January 1, 2022. The primary outcome was the response rate to immunosuppressive therapy for ILICI, with subgroup analysis based on the type of cancer, immune checkpoint inhibitor regimen, and severity of liver injury. The secondary outcome was the median time to recovery from ILICI with immunosuppressive therapy.

**Results:**

A total of 30 studies that included 1120 patients were collected. The pooled ILICI response rate was 79% (95% CI 0.73-0.84) for treatment with corticosteroids and 93% (95% CI 0.79-1.0) for treatment with mycophenolate mofetil. For ILICI treated with corticosteroids, the median recovery time was 47.59 (95% CI 39.79-55.40) days compared to 37.74 (95% CI 31.12-44.35) days for all forms of immunosuppression.

**Conclusion:**

Findings support the effectiveness of corticosteroids and mycophenolate mofetil for the treatment of ILICI. The identified median time to recovery is a beneficial guide for patients and physicians, allowing for realistic expectations and appropriate treatment management. Future prospective randomized controlled trials are required to define a standardized management approach to immunosuppressive therapy of ILICI.

**Systematic review registration:**

https://www.crd.york.ac.uk/PROSPERO/, identifier CRD42022313454.

## 1 Introduction

The advent of immune checkpoint inhibitors (ICIs) has recently altered the landscape of conventional cancer treatment, improving prognosis and remission rate (1). ICIs are monoclonal antibodies that target programmed cell death protein 1 (PD-1)/programmed death-ligand 1 (PD-L1) and cytotoxic T-lymphocyte-associated antigen 4 (CTLA-4), which reinvigorate T cell responses to tumor cells (2). However, given the increased use of immunotherapy and a shift in the exploration of combination regimens, clinical safety concerns regarding the use of ICIs have arisen. Further, immune-related adverse events (irAEs) have to some extent hindered the implementation of ICIs in clinical practice (3).

Immune-mediated liver injury caused by checkpoint inhibitors (ILICI) is a common irAEs whose pathogenesis and clinical features have not yet been fully elucidated (4–6). Several meta-analyses have shown that the incidence of ILICI ranges from 2% to 30% (7–9). According to previous studies, a few factors have been illustrated to influence the risk of ILICI development or patterns of ILICI, such as the type, dose, or duration of immunotherapy (10, 11). ILICI clinical presentation is extremely heterogeneous, from asymptomatic elevations of liver enzymes to, more rarely, severe fulminant hepatitis and liver failure (9, 12). Clinical treatment is based primarily on expert consensus guidelines, which recommend corticosteroids and other second-line immunosuppressive agents such mycophenolate mofetil (MMF), tacrolimus, and cyclosporine (13). However, some patients exhibit spontaneous improvement while others develop resistance to corticosteroids or MMF, necessitating the initiation of multiple regimens or even plasma exchange (14, 15).

Currently, there are no biomarkers that predict treatment outcomes for immune-related liver damage. Further, there is little available guidance, from randomized controlled trials, regarding the best course for effective treatment (9). Even though corticosteroids and other immunosuppressants have been extensively used to manage ILICI, their effectiveness is not fully established. It is acknowledged that conduct of appropriate prospective clinical trials is a challenge. Therefore, the purpose of this study was to pool immunosuppressant response rates and median time to recovery for patients with ILICI. In this manner, empirical adoption of immunosuppressants for treatment and management of ILICI was evaluated and effectiveness determined.

## 2 Materials and methods

### 2.1 Data source and search strategy

This study was performed following the Preferred Reporting Items for Systematic Reviews and Meta-Analyses (PRISMA) guidelines (16) and was prospectively registered with the International Prospective Register of Systematic Reviews (Registration number: CRD42022313454). To identify eligible literature, we conducted a systematic search in PubMed, Ovid, and Cochrane Library from 2000 to January 1, 2022, with MESH terms and free text including ‘immune checkpoint inhibitor’, ‘Programmed cell death (Ligand) 1’, ‘cytotoxic T lymphocyte antigen 4’ as well as its specific names AND ‘immune-related Hepatotoxicity’, ‘immune induced liver injury’ (detailed search strategy is found in **Supplementary 1**).

### 2.2 Inclusion and exclusion criteria

The following pre-determined inclusion criteria were used: (i) Patients with any cancer receiving immune checkpoint inhibitor treatment and developing ILICI; (ii) Immunosuppressants (including corticosteroids) were administrated for ILICI; and (iii) Available data for accessing response or recovery median time of immunosuppressants therapy. The exclusion criteria were as follows: (i) Outcomes of interest were ambiguous for analysis; (ii) The number of immunosuppressants-treated patients was fewer than 5; and (iii) Combination with additional techniques (such as stereotactic body radiotherapy, radiofrequency ablation).

### 2.3 Data extraction and assessment of study quality

Two independent study investigators (Chen K and He J) reviewed the included articles and extracted relevant information based on a predefined protocol. Disagreements were settled *via* consensus. The data extraction form included the following details about the studies: author, year, study design, and baseline characteristics (immune checkpoint inhibitor regimens, underlying cancer, and the number of patients who developed ILICI). Other data extracted were available for regimens and duration of corticosteroids as well as other regimens used for corticosteroids refractory cases. The primary outcome was the number or proportion of patients responding to immunosuppressive therapy for ILICI. The secondary outcome was time from the onset to ILICI improvement or resolution. We collected response rates to corticosteroids and mycophenolate mofetil as defined by the authors of each study. For studies without provided corticosteroid responsiveness, patients who needed second-line immunosuppressants were treated as lacking response to corticosteroids based on the definition of steroid-refractory (17). Study eligibility was accessed independently by two authors using a quality appraisal tool that covers an 18-point checklist that evaluated the quality of selected studies that consisted of the following seven aspects: study objective, study population, intervention and co-intervention, outcome measures, statistical analysis, results, and conclusions, competing interests, and source of support (18). Cut-off values were used to categorize the identified studies into high (13-18 points), moderate (7-12 points), and low quality (0-6 points). All emerging conflicts were resolved after discussion.

### 2.4 Data synthesis and statistical analysis

Meta-analysis was structured around two parts. For the first part, a random-effects model (19) was used to assess the pooled estimated proportion of patients who responded to corticosteroids, while a common effect model was used for those who responded to MMF depending on the between-study heterogeneity in effect size. Raw data were transformed due to poor mathematical properties. Freeman-Tukey Double arcsine transformation was applied to corticosteroid and MMF effect calculations in order to improve the reliability of parameter estimation. Moreover, subgroup analyses were carried out according to the type of cancer, immune checkpoint inhibitor regimens, and grade of ILICI. If a study could not be assigned to any specific subgroup, it was classified into a “mixed” group. For the second part, time to resolution with immunosuppressant treatment was assessed, using the median as the effect size. We employed weighted median time to resolution from onset coupled with its corresponding 95% confidence interval (95% CI) as summary statistics using the method described by McGrath et al. (20). All statistical analyses were performed with the statistical software R version 4.1.2 (The R Foundation for Statistical Computing, Vienna, Austria) [package “meta v5.2-0”, package “metamedian v0.1.5”]. Overall combined results, derived from aggregating the individual studies, were used to generate forest plots.

Cochrane’s Q and I-squared (*I^2^*) tests were used to assess statistical heterogeneity among enrolled studies with a threshold *I^2^* value of 0-40%, 30-60%, 50-90%, and 75-100% to indicate not important, moderate, substantial, and considerable heterogeneity according to the Cochrane Handbook. Publication bias was evaluated by visual inspection of funnel plots and Egger’s linear regression (21), which statistically examines the asymmetry of funnel plots. Statistical significance was a *p*-value of 0.05 or below.

## 3 Results

### 3.1 Eligible studies and characteristics

**Figure 1:**
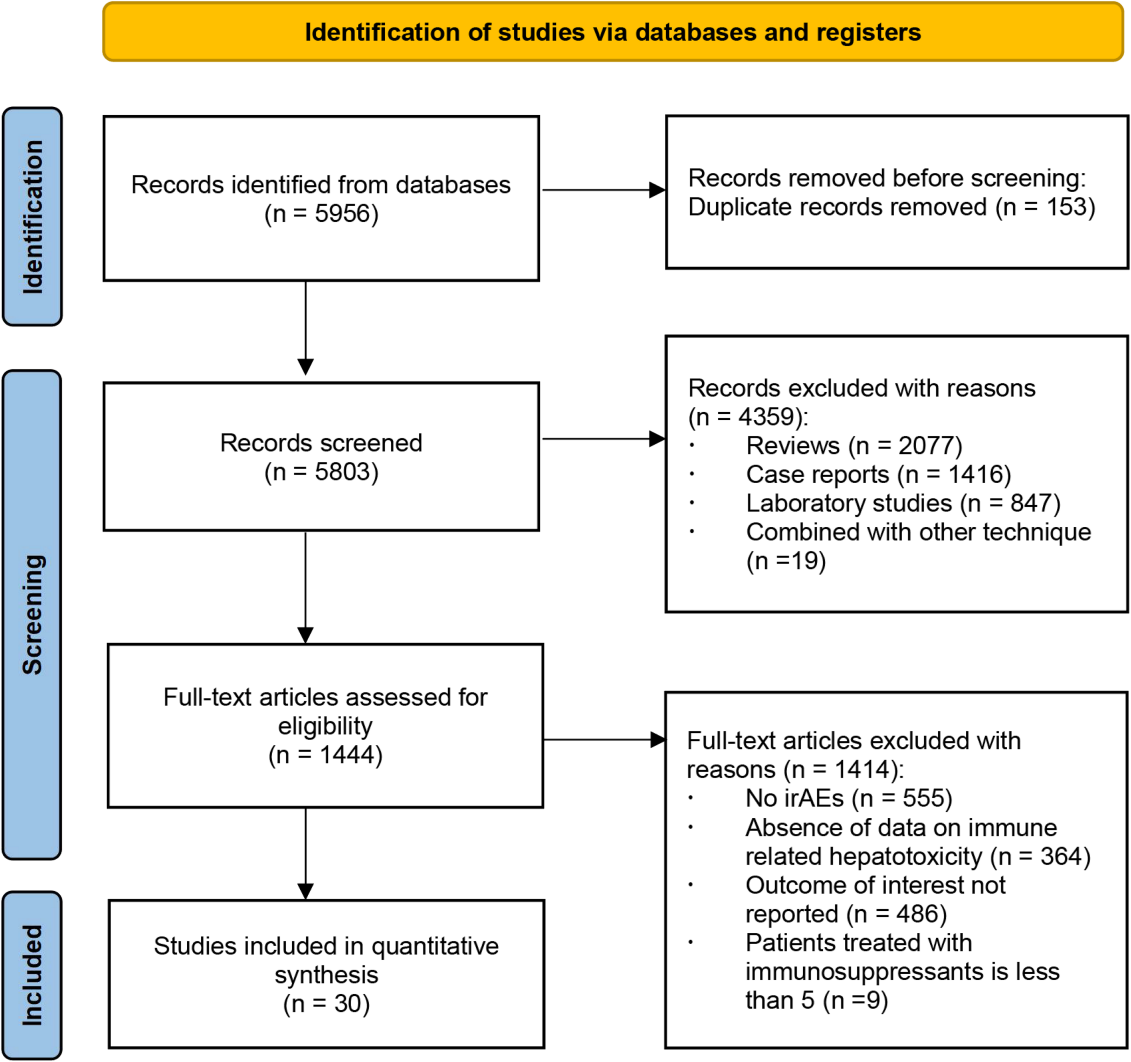
Flow diagram of the study selection process.

Overall, we initially retrieved a total of 5956 records from databases, which were narrowed down to 1444 after a screening of abstracts. Of the remaining, 1414 studies were excluded for a variety of reasons. We identified 30 eligible studies with a total of 1120 patients providing response to immunosuppressant data. The detailed selection process is listed in **Figure 1** with the main characteristics of the included studies outlined in **Table 1**. It’s apparent that the number of included studies increased significantly from 2020 to 2021, 14 of which were published in 2021 and 7 in 2020. Studies consisted mainly of retrospective cohort studies (n = 25), followed by case series (n = 3), prospective cohort (n = 1), and pooled analysis (n = 1).

**Table 1:** Characteristics of the studies included in the meta-analysis.

Study	Baseline characteristics			ILICI		Management of ILICI							
	Design	Type(s) of cancer	Treatment regimen(s)	Diagnosis and Grade	No. of ILICI (G1-G2/G3-G5)	CS use n(%)	CS regimen(s)	CS response n(%)	Duration of CS Median (IQR)	MMF use n(%)	MMF response n(%)	Time to ①resolution/②improvement to G1 ILICI	Other regimen(s) used in CS refractory
Purde et al. 2021 (22)	Prospective cohort, Single-center	Melanoma, NSCLC	Anti-PD-1/L1, Anti-CTLA-4, Combination	LFT, R value, CTCAE V5.0	11 (6/5)	6/11 (55%)	/	6/6 (100%)	80 (10-145.5)D	/	/	/	/
Patrinely et al. 2021 (23)	Retrospective cohort, Multi-center	Lung cancer, Melanoma, RCC, SCC, Others	Anti-PD-1/L1, Anti-CTLA-4, Combination	LFT, Symptoms, Biopsy, Imaging, CTCAE V5.0	164 (66/98)	150/164 (91%)	Prednisone or mPSL (>1mg/kg, PO or IV)	113/150 (75%)	/	34/37 (92%)	/	①Median: 52D ②Median: 13D	MMF+ Tacrolimus (3), MMF + Abatacept (1), Infliximab (1)
Cunningham et al. 2021 (24)	Retrospective cohort, Single-center(Phase I or II clinical trial)	Melanoma, Head and neck, Genitourinary, Lung, Gastrointestinal	Anti-PD-1/L1, Anti-CTLA-4, Combination	LFT ≥ G2, RUCAM, R ratio, Diagnosis of exclusion, CTCAE V5.0	17 (4/13)	15/17 (88%)	Dexamethasone 4mg, Steroid 1-2mg/kg IV, Prednisone 1mg/kg	14/15 (93%)	/	1/1 (100%)	1/1 (100%)	①Median(Range): 43(12-102)D	/
Ito et al. 2021 (25)	Retrospective cohorts, Multi-center	Lung cancer, Melanoma, RCC, Head and neck carcinoma, Others	Anti-PD-1/L1, Anti-CTLA-4, Combination	LFT ≥ G3, Diagnosis of exclusion, CTCAE V5.0	58	30/58 (52%)	G3:Prednisone 0.5–1.0 mg/kg/d PO; G4: Steroid + mPSL + Prednisone(1.0–2.0 mg/kg/d)	19/30 (63%)	/	3/11 (27%)	1/3 (33%)	/	UDCA only(3) with resolution(2), PSL+UDCA(8)
Li et al. 2021 (26)	Retrospective cohort, Single-center	Melanoma, NSCLC, RCC, Breast cancer, Others	Anti-PD-1/L1, Anti-CTLA-4, Combination	ALT > 200 U/L, Diagnosis of exclusion, Appropriate response to corticosteroids	201 (NA/201)^1^	106/106 (100%)^2^	Maximum CS doses: Median(IQR) 1.5(1.0-2.0)mg/kg/d	88/106 (83%)	/	/	/	①Median(IQR): 33(28-39)D ②Median(IQR): 15(14-17)D	/
Li et al. 2021 (27)	Retrospective cohort, Multi-center	Melanoma, NSCLC, RCC, Breast cancer, Urothelial cancer, Others	Anti-PD-1/L1, Anti-CTLA-4, Combination	ALT > 200 U/L, Hepatocellular pattern of injury, Diagnosis of exclusion, CTCAE V5.0	215 (NA/215)	215/215 (100%)	Initial steroid dose Lower-dose groups: Median(IQR) 0.8(0.8-1.0)mg/kg Higher-dose groups: Median(IQR) 2.0(2.0-2.0)mg/kg	154/215 (72%)	Higher-dose groups: 60(40-85)D Lower-dose groups: 44(32-70)D	/	/	①Median: Higher-dose groups: 29D Lower-dose groups: 28D ②Median: Higher-dose groups: 15D Lower-dose groups: 14D	/
Takinami et al. 2021 (28)	Retrospective cohort, Single-center	NSCLC, Melanoma, RCC	Anti-PD-1, Anti-CTLA-4, Combination	AST or ALT or ALP ≥ G2, RUCAM, CT scan, Diagnosis of exclusion, CTCAE V5.0	41(28/13)	7/12 (58%)^3^	/	4/7(57%)	/	2/3 (67%)	2/2 (100%)	②Median(IQR) irH: 36(25-54)D irSC: 102.5(72-134)D	/
Yamamoto et al. 2021 *(* 29)	Retrospective cohort, Single-center	NSCLC, RCC, Urothelial cancer, Melanoma, Others	Anti-PD-1/L1, Anti-CTLA-4, Combination	AST or ALT ≥G2 Diagnosis of exclusion, CTCAE V5.0	21 (7/14)	13/21 (62%)	Steroids 0.5-1mg/kg	7/13 (54%)	/	2/6 (33.3%)	2/2 (100%)	/	UDCA only(5) with resolution(5), Steroids+UDCA(4) with resolution(4)
Robert et al. 2021 (30)	Pooled analysis of 3 clinical trials	Melanoma	Anti-PD-1	Based on mechanism of action and a prespecified list of terms, CTCAE V4.0	23	17/23 (74%)	/	16/17 (94%)	/	/	/	①Median(Range) 8.6(0.6-26.1)W	/
Luo et al. 2021 (17)	Retrospective cohort, Single-center	Lung cancer	Anti-PD-1/L1, Combination	CTCAE V5.0	6	/	/	/	/	6/6 (100%)	5/6 (83%)	/	/
Biewenga et al. 2021 *(* 31)	Retrospective cohort, Multi-center	Melanoma	Anti-PD-1, Anti-CTLA-4, Combination	AST or ALT ≥G3 CTCAE V4.0	139 (NA/139)	123/124 (99%)^4^	/	98/123 (80%)	/	/	/	/	Unspecified(25)
Samanci et al. 2021 (32)	Retrospective cohort, Single-center	Breast cancer, Bladder Cancer, Stomach cancer, RCC	Anti-PD-1/L1, Combination	CTCAE V4.0	13 (8/5)	5/5 (100%)^5^	IV steroids first followed by PO steroids	4/5 (80%)	/	1/1 (100%)	1/1 (100%)	/	/
Cohen et al. 2021 (33)	Retrospective cohorts, Single-center	Melanoma, NSCLC, Gastrointestinal tumors, Others	Anti-PD-1/L1, Anti-CTLA-4, Combination	LFT, Biopsy, CTCAE	60 (7/53)	51/60 (85%)	/	39/51 (76%)	/	/	/	②Median(Range): 52(2–302)D	Non-specific secondary immunosuppression(10), Infliximab(2)^6^
Gauci et al. 2021 (34)	Retrospective cohort, Single-center	Melanoma	Anti-PD-1, Anti-CTLA-4, Combination	LFT, Biopsy, ADR probability scale, Diagnosis of exclusion, CTCAE V4.03	21 (NA/21)	13/21 (62%)	Steroids: Median(range): 1(0.3-2)mg/kg/d	12/13 (92%)	1.8(1.7-3.5)M	/	/	Median: 49D	/
Riveiro-Barciela et al. 2020 (35)	Retrospective cohorts, Single-center	NSCLC, Melanoma, Urothelial cancer, Others	Anti-PD-1/L1, Anti-CTLA-4, Combination	ALT ≥ G3, Diagnosis of exclusion, RUCAM, CTCAE V4	28 (NA/28)	28/28 (100%)	Median CS doses: 60 mg/d	18/28 (64%)	2.3 (1.3-3.1)M	10/10 (100%)	9/10 (90%)	①Median(IQR): 1.5 (0.7-4.15)M	Plasma exchange(1)
Miller et al. 2020 (36)	Retrospective cohort, Single-center	Melanoma, Genitourinary, Lung cancer, Head and neck cancer, Gastrointestinal cancer, Others	Anti-PD-1/L1, Anti-CTLA-4, Combination	ALT ≥ 5 × ULN, Diagnosis of exclusion, Clinical signs and symptoms, CTCAE V4.03	100 (NA/100)	67/100 (67%)	/	64/67 (96%)	43(25-90)D	3/3 (100%)	3/3 (100%)	②Median(IQR) 23(14-35)D	/
Li et al. 2020 *(* 37)	Retrospective cohort, Multi-center	Melanoma	Anti-PD-1/L1, Anti-CTLA-4, Combination	ALT ≥200 U/L, Diagnosis of exclusion, CTCAE V5.0	102 (NA/102)	97/102 (95%)	Systemic CS therapy at a dose of at least 1 mg/kg prednisone equivalents	63/97 (65%)	/	32/34 (94%)	/	①Median(IQR): Rechallenge & No discontinuation Group (N=25): 30(21-55)D Rechallenge & discontinuation Group (N=6): 5(21-40)D	AZA (2)
Zhang et al. 2020 (38)	Retrospective case series, Multi-center	Melanoma, Pancreatic adenocarcinoma, Head and neck, Ovarian carcinoma, Refractory Hodgkin lymphoma	Anti-PD-1	LFT, Biopsy	8	8/8 (100%)	/	5/8 (62.5%)	/	3/3 (100%)	3/3 (100%)	/	/
Romanski et al, 2020 *(* 39)	Retrospective cohort, Single-center	Melanoma	Anti-PD-1, Anti-CTLA-4, Combination	ALT, AST and/or TBili ≥ G2, CTCAE V5.0	222 (194/28)	31/43 (72%)^7^	Cumulative dose of PSL: Median(range) Grade 2: 737.5(375-6000)mg Grade 3: 2325(575-5987.5)mg Grade 4: 4975(1867.5-6000)mg	29/31(94%)	/	2/2 (100%)	2/2 (100%)	①Median(Range): 48(12-80)D	/
Mizuno et al. 2020 (40)	Retrospective cohort, Multi-center	Lung cancer, Melanoma, Head and neck carcinoma, RCC, Gastric carcinoma, Others	Anti-PD-1/L1, Anti-CTLA-4	LFT, CT, MRI, R value, Diagnosis of exclusion, CTCAE V4.03	29 (NA/29)	16/29 (55%)	/	8/16 (50%)	/	3/8 (37.5%)	/	/	PSL+UDCA(5) PSL+MMF+UDCA(2)
Zen et al. 2020 (41)	Retrospective cohort, Multi-center	NSCLC, Urothelial carcinoma, Merkel cell carcinoma, Melanoma, Colon cancer	Anti-PD-1/L1	Blood test results, R score, Biopsy	10	10/10 (100%)	PSL40-80mg/d, mPSL 500 mg/d	7/10 (70%)	/			①Median(Range): 4(2-12)W	PSL+MMF+AZA(1)
Imoto et al. 2019 (42)	Retrospective cohort, Single-center	Lung cancer, Melanoma, Head and neck cancer, Renal cancer, Stomach cancer, Others	Anti-PD-1/L1, Anti-CTLA-4, Combination	LFT, Biopsy Full-liver screening tests, CTCAE V4.0	56 (45/11)	6/53 (11%)^8^	PSL 100mg/d PSL 0.6mg/kg/d mPSL 1000mg/d	3/6(50%)	/	2/3 (67%)	/	/	Infliximab^9^(1), UDCA(2), UDCA+mPSL(1)
Cheung et al. 2018 (43)	Retrospective cohort, Single-center	Melanoma, Lung cancer, RCC, Epithelial Mesothelioma	Anti-PD-1, Anti-CTLA-4, Combination	CTCAE V5.0	21(7/14)	19/21 (90%)	mPSL IV or PSL PO	9/19 (47%)	/	8/10 (80%)	6/8(75%)	/	Infliximab+mPSL(1) resolution, Infliximab+PSL+mPSL+MMF(1) resolution, PSL+MMF+tacrolimus(1) not resolution, UDCA+PSL(1)resolution
Martin et al. 2018 (44)	Retrospective cohort, Single-center	Melanoma, Bronchial carcinoma, Renal clear cell carcinoma, Bladder carcinoma, Cholangiocarcinoma	Anti-PD-1/L1, Anti-CTLA-4, Combination	DILIN 5-point scale, RUCAM, CTCAE V4.03	16 (NA/16)	10/16 (62.5%)	CS at 0.2, 0.5–1, 2.5 mg/kg/d	9/10 (90%)	/	1/1 (100%)	/	/	/
Gauci et al. 2018 (45)	Retrospective cohort, Single-center	Melanoma	Anti-PD-1, Anti-CTLA-4, Combination	/	10(1/9)	5/10 (50%)	Steroids 0.3, 1 or 2mg/kg/d	4/5(80%)	/	/	/	①Median(Range): 8.6(4.3-55.1)W	UDCA+Steroids(1), resolution
Huffman et al. 2018 (46)	Retrospective cohort, Single-center	Melanoma	Anti-PD-1, Anti-CTLA-4, Combination	AST, ALT and/or TBili CTCAE V4.0	17 (4/11)^10^	16/17 (94%)	Prednisone 1-2 mg/kg, mPSL 1g/d, Dexamethasone	14/16 (87.5%)	Median(Range): 42(7-78)D	/	/	①Median(Range): 31(6-56)D	AZA+PSL(1),not improvement, Cyclosporine + PSL(1),improvement
Hofmann et al, 2016 (47)	Retrospective cohort, Multi-center	Melanoma	Anti-PD-1	CTCAE V4.0	11 (NA/11)	11/11 (100%)	PSL or mPSL 1-2 mg/kg PO or IV	8/11 (73%)	/	3/3 (100%)	3/3 (100%)	/	/
Horvat et al. 2015 (48)	Retrospective cohort, Single-center	Melanoma	Anti-CTLA-4	CTCAE V4.0	197 (158/39)	22/197 (11%)	/	20/22 (91%)	/	2/2 (100%)	/	/	/
Johncilla et al. 2015 (49)	Retrospective case series, Multi-center	Melanoma	Anti-CTLA-4	LFT, Biopsy	11	11/11 (100%)	Prednisone and solumedrol	8/11 (73%)	/	2/3 (67%)	2/2 (100%)	Time to improvement or resolution: within 2W-3M	6-Mercaptopurine + PSL(1) , remission
Kim et al. 2013 (50)	Retrospective case series, Single-center	Melanoma	Anti-CTLA-4	LFT, CT, Symptom	6	6/6 (100%)	Solumedrol 120 mg/kg IV, PSL PO, Tapering over 2–6 months	6/6 (100%)	/	/	/	①Median(IQR): 77.5(44.5-120.5)D	/

^1^ Including Biopsy + ILICI: 95 cases; Non-biopsy + ILICI: 106 cases.

^2^ Only calculated non-biopsy + ILICI due to data availability.

^3^ 12 cases with CT due to data availability.

^4^ For 15 episodes treatment details were not registered.

^5^ Only include ≥ Grade 3.

^6^ Infliximab was used for the treatment of concurrent immune-induced colitis.

^7^ Only include ≥ Grade 2.

^8^ 3 deaths for underlying disease.

^9^ Infliximab was used for the treatment of checkpoint inhibitor-induced pneumonitis.

^10^ 2 patients had initial lab data unavailable to be graded.

NSCLC, Non-small cell lung carcinoma; RCC, Renal cell carcinoma; SCC, Squamous cell; carcinoma; LFT, Liver function test; ULN, Upper limit of normal value; CS, Corticosteroids; MMF, Mycophenolate mofetil; PSL, Prednisolone; mPSL, Methylprednisolone; UDCA, Ursodeoxycholic acid; AZA, Azathioprine; irH, Immune-related hepatitis; irSC, Immune-related sclerosing cholangitis.

In the majority of the included studies, diagnosis and grade of ILICI were defined in accordance with CTCAE version 4.0 (n = 8), 4.0.3 (n = 4), and 5.0 (n = 11), where the elevation of liver enzymes is the most common clue for ILICI. Due to its invasive nature, only seven reported outcomes for diagnostic liver biopsy. Other methods of diagnosis were; CT scan, MRI, and scales such as RUCAM and the ADR probability scale. Most ILICI occurred within 1 to 3 months of ICI initiation. In addition to corticosteroids that have been widely used for treatment of ILICI, adequate data for treatment with MMF were also available and included in the pooled response rate for this study. However, alternative immunosuppressants including azathioprine, tacrolimus, infliximab, and anti-thymocyte globulin administered in steroid-refractory cases, were not included due to the limited available data. Several studies recorded time to resolve or improve Grade 1 ILICI subsequent to immunosuppressants, which were also treated as a sign of effective treatment.

### 3.2 Efficacy of corticosteroids

A total of 29 studies involving 1114 patients were available for calculation of corticosteroid responsiveness. In the majority of studies, more than half of the patients required corticosteroids, but the usage remained different dependent upon the study population. Imoto et al. (42) and Horvat et al. (48) reported an 11% utilization of corticosteroid therapy in patients with low-grade ILICI. Approximately 80% of the patients in both studies improved spontaneously without corticosteroids.

A total of 11 studies mentioned the specific steroids used (23–25, 37, 41–43, 46, 47, 49, 50), wherein, prednisone and methylprednisolone were used in nine studies (23, 25, 41–43, 46, 47, 49, 50), with only two studies mentioned individuals treated with dexamethasone (24, 46). Common routes of treatment were oral and injection. In most of the studies, mean dose of corticosteroids was reported, whereas Li et al. and Romanski et al. provided the maximum dose (26) and cumulative dose (39), respectively. The therapeutic dose of steroids varied from 0.2 to 2 mg/kg, generally based on the severity of lLICI and the treatment experience of physicians, combined with therapeutic management guidelines.

**Figure 2:**
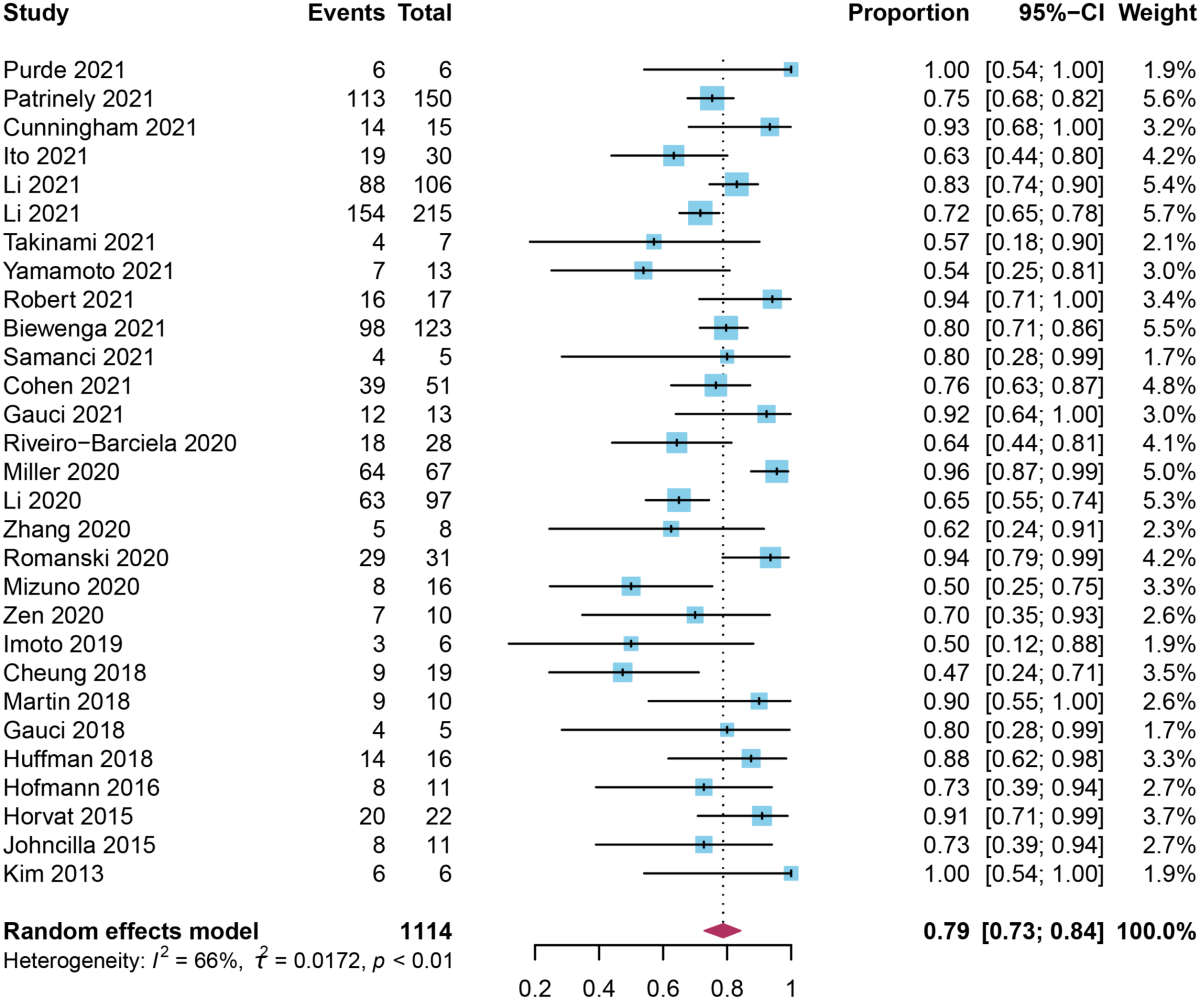
Forest plot of pooled response rate to corticosteroids in patients with checkpoint inhibitor-induced liver injury.

Duration for corticosteroids therapy was reported for five studies (22, 27, 34–36), with a median period of 42 to 80 days. The pooled result was 51.34 (95% CI: 41.87-60.81) days, with 69% of *I^2^* statistic, indicating a significant heterogeneity (**Supplementary Figure 1**). The pooled response to corticosteroids was 79% (95%CI 0.73-0.84), which was associated with a high degree of significant heterogeneity (*I^2^* = 65.9%, *p* < 0.0001) (**Figure 2**).

We conducted subgroup analysis according to the type of cancer, treatment regimen, and grade of ILICI. No statistically significant difference was found among groups or among the three subgroups (Type of cancer: *P_subgroup_* = 0.07; Treatment regimen: *P_subgroup_* = 0.40; Grade of ILICI: *P_subgroup_* =0.72; **Table 2**, **Supplementary Figures 2**–**4**). For subgroup analysis of cancer type, there were 11 studies involving melanoma with greater treatment response rates than overall 85% (95% CI 0.77-0.92) *vs.* 79% (95% CI 0.73-0.99). To assess the response rate to different immune-checkpoint inhibitor regimens, including anti-PD-(L)1 and anti-CTLA-4, another subgroup analysis was carried out. The pooled response rate in the group with anti-CTLA-4 was higher than the group with anti-PD-(L)1 0.90 (95%CI 0.74-0.99) *vs.* 0.79 (95%CI 0.63-0.92). With regard to the grade of ILICI, the treatment response was somewhat less effective in cases of high-grade liver injury compared to the overall response rate 77% (95% CI 0.68-0.85) *vs.* 79% (95% CI 0.73-0.99).

**Table 2:** Subgroup analysis based on the type of cancer, treatment regimen, and grade of ILICI.

	Proportion	95%-CI	*τ^2^*	*I^2^*	*p*	*P _subgroup_*
**Type of cancer**						0.07
** Mixed**	0.74	[0.66; 0.82]	0.0203	70%	<0.01	
** Melanoma**	0.85	[0.77; 0.92]	0.0115	57%	<0.01	
**Treatment regimen**						0.40
** Mixed**	0.77	[0.70; 0.84]	0.0199	72%	<0.01	
** Anti-PD-(L)1**	0.79	[0.63; 0.92]	0.0090	16%	0.31	
** Anti-CTLA-4**	0.90	[0.74; 0.99]	0.0055	27%	0.25	
**Grade of ILICI**						0.72
** All-grade**	0.80	[0.71; 0.88]	0.0193	55%	<0.01	
** High-grade**	0.77	[0.68; 0.85]	0.0176	76%	<0.01	

### 3.3 Efficacy of mycophenolate mofetil

**Figure 3:**
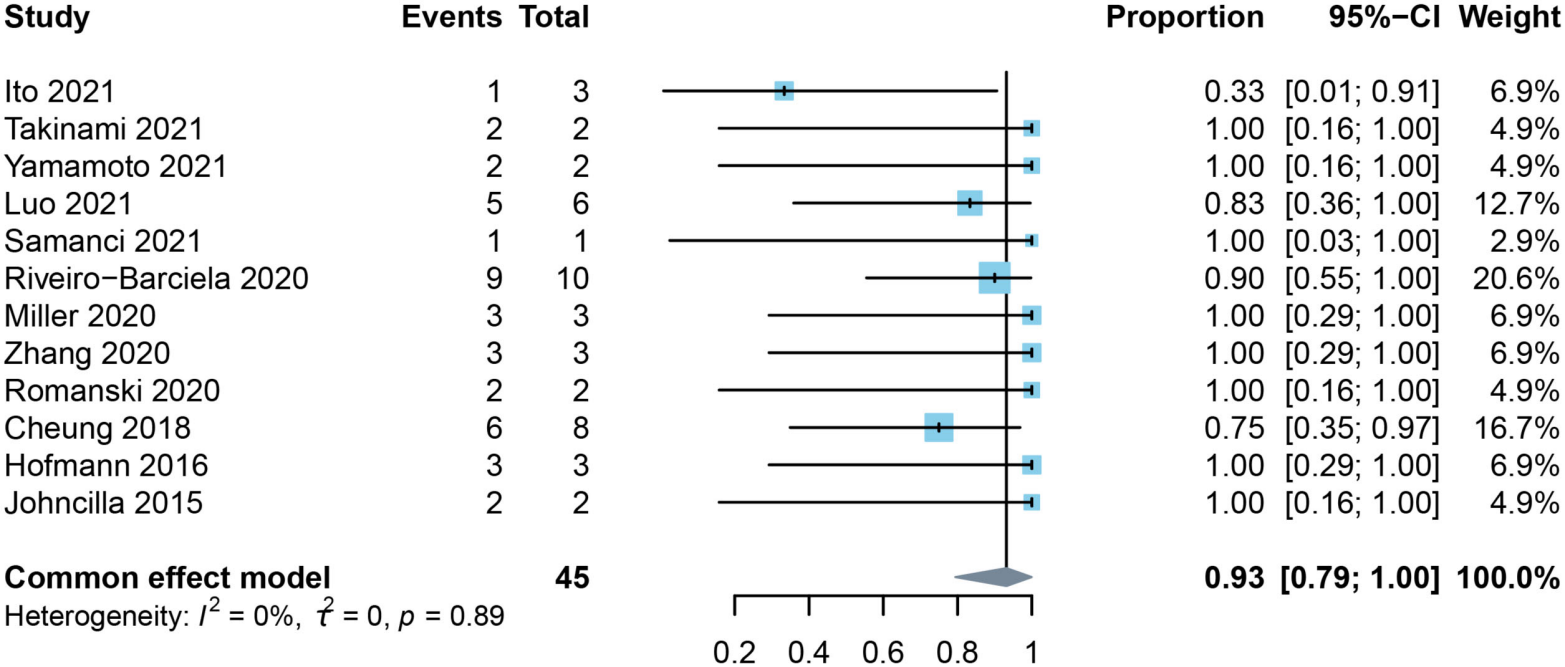
Forest plot of the pooled response rate to mycophenolate mofetil in patients with checkpoint inhibitor-induced liver injury.

There were 12 studies that contained data for estimation of response rates to MMF. Most of these studies included a range of underlying cancers as well as the administration of different types of immune checkpoint inhibitors. The study by Luo et al. (17) focused on lung cancer, Romanski et al. (39) concentrated on melanoma, and Zhang et al. (38) limited their study to PD-1 inhibitors. Seven of the included studies reported that MMF was selected for post-line therapy in all steroid-refractory or resistant cases. Our pooled result showed that MMF achieved a 93% (95% *CI*: 0.79-1.0) response rate for the treatment of ILICI with no heterogeneity (*I^2^* = 0%) (**Figure 3**).

Luo et al. specifically discussed the efficacy of second-line immunosuppressive therapy for steroid-refractory or resistant immune-related adverse events. In that study, five of six patients (83%) with immune-related hepatitis improved within 90 days after receiving MMF for a median of three months (range: 2-5 months) (17). They also reported that a patient receiving infliximab for colitis died from biopsy-diagnosed infliximab-associated hepatotoxicity. Miller et al. described three patients who had progressive worsening of ALT despite corticosteroid use, which led to the introduction of MMF. Two individuals were administrated MMF after four weeks and one after two weeks (36). After initiation of MMF, ALT decreased to grade 1 or lower within 10 days in one patient and to grade 1 within 20 days in the other two patients. The general dose for MMF, in that study, was 500 mg or 1.0 g bid, which was in compliance with the recommendation of the guidelines.

### 3.4 Time to resolution with immunosuppressant treatment

A total of 17 studies reported time to recovery following immunosuppressive therapy for ILICI. Some studies described time to improve to grade 1 after treatment while others examined the time to return to normal. In this analysis, we synthesized the time to return to normal with immunosuppressive treatment for a total of nine studies. Median time was 37.74 (95% CI 31.12-44.35) (**Figure 4A**) days with moderate heterogeneity (*I^2^* = 58.28%, *p* = 0.0171). Four studies reported a median time to normal recovery with corticosteroids of 47.59 (95% CI 39.79-55.40) (**Figure 4B**) days with no observed heterogeneity (*I^2^* = 0%).

**Figure 4:**
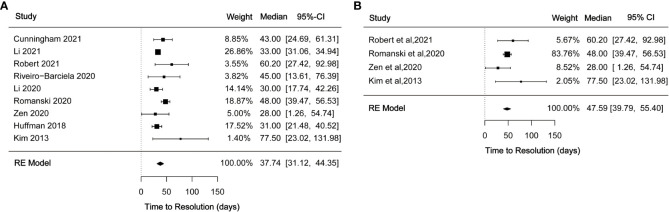
**(A)** Forest plot of time to resolution with immunosuppressant treatment; **(B)** Forest plot of time to resolution with corticosteroid treatment.

### 3.5 Study quality and publication bias

With regard to quality assessment, study scores ranged from 8 points to 15 points with an average of 11.5 points. Eight studies were deemed to be of high quality, with the remaining categorized as moderate quality (**Supplementary Table 1**). Reduced quality scores were due to a lack of adverse event reporting, loss to follow-up, poor description of participant study entrance with regard to disease state, and lack of random variability estimates. Studies that concentrated on other main outcomes such as liver biopsy (33), histologic patterns of ILICI (38), and immunosuppressive management consequences frequently lacked sufficient detail, which resulted in a lower quality assessment.

Funnel plots presented no statistically significant asymmetry regarding the effectiveness of corticosteroids and MMF, with *p*-values of 0.8187 (**Supplementary Figure 5A**) and 0.4465 (**Supplementary Figure 5B**) for Egger’s test, respectively. These results indicate no publication bias. Funnel plots and Egger’s test were not performed for time to resolution in that the number of included studies was insufficient (less than 10).

## 4 Discussion

ILICI exhibits heterogeneity in clinical presentation, management, and treatment outcomes, with guidelines for immunosuppressant management of ILICI controversial (44, 45). Thus, the knowledge base for ILICI immunosuppressant use is limited with adverse events significant and the overall impact on cancer immunotherapy unknown (51). As such, we conducted a meta-analysis and a systematic review to comprehensively evaluate immunosuppressive agent response rates for ILICI in order to provide for a benefit assessment of immunosuppressant management of ILICI. A key finding was that immunosuppressant showed favorable clinical outcomes for ILICI.

Corticosteroids are the cornerstone of treatment for ILICI but the current recommendations for ILICI management are based on a colitis model (45, 52). It is important to note that corticosteroids are more effective for the treatment of ILICI than for immune-associated colitis in which the pooled response rate was 59% by meta-analysis (53). Future studies into the underlying molecular basis for ILICI are needed so that appropriate therapeutic strategies can be implemented for management of ILICI.

Differences in the incidence and severity of ILICI mainly depend upon the type of cancer and immune checkpoint inhibitor regimens employed (54). It is unknown whether a lower rate of response to corticosteroids is found with more severe liver injury, which prompted us to conduct subgroup analysis of underlying cancer, immunotherapy regimen, and ILICI grade. Even though statistically significant results were not obtained, potential trends were identified. Previously, disease-specific clinical factors have been suggested to impact corticosteroid therapy outcomes, although the low frequency of ILICI for some malignancies limits response rate evaluation of various underlying tumors. The effectiveness of corticosteroids for the group of melanoma patients was higher than the overall average, which suggests the effectiveness of corticosteroids for such patients. Regarding therapeutic regimens, existing studies have shown that anti-CTLA-4 is a risk factor for immune-related liver injury (7), but whether it is also a predictor of corticosteroid responsiveness requires further exploration. The response rate to corticosteroids for patients treated with anti-CTLA-4 was higher than that of patients treated with anti-PD-1/L1, although statistical significance was not achieved. Further study is required to clarify this result. With regard to severe liver injury, treatment recommendations for Grade 2 ILICI differ between guidelines: SITC and NCCN recommended initiation of steroid therapy, whereas ASCO and EMSO advise liver enzyme monitoring before steroid therapy, if abnormal liver function persists for 3-5 days without improvement. For grade 3 ILICI, De Martin et al. demonstrated that the administration of corticosteroids should be based on liver parameters including PT values, bilirubin levels, and the severity of histological damage (44). In the Romanski et al. study, 40% of 15 patients with grade 2 ILICI received steroids, with only one patient experienced hepatitis recurrence during treatment. In contrast, 87% of 23 patients with grade 3 ILICI received steroids, and of those 8 patients (40%) relapsed during treatment, showing that the response to corticosteroids depends upon the severity of liver injury (39).

A variety of corticosteroid dosages (average, maximum, maintenance, and cumulative), as well as diverse specific corticosteroids (prednisone, methylprednisolone, and dexamethasone), were reported in the include studies, which made conduct of a quantitative analysis challenging for assessment of the effectiveness of various corticosteroid regimens for ILICI. Whether or not a particular corticosteroid regimen is more effective than another requires further investigation. Corticosteroids are recommended for immune-related hepatotoxicity at a dose of 0.5-2 mg/kg/day (52, 55, 56), with physicians still exploring best practice. In a single-center retrospective cohort study, improved liver function tests were observed in three patients with grade 3-4 ILICI who had not received corticosteroids, and two who received 0.6 mg/kg of prednisolone, indicating that corticosteroid therapy may not always be necessary (42). Similar conclusions were found in another study in which a lower dose of 50-60 mg of prednisolone had clinical benefit (43). Compared with a high-dose regimen, initial treatment with methylprednisolone 1 mg/kg/day provided similar outcomes and reduced the risk of steroid-related complications for severe ILICI (27). These results challenge existing guidelines in terms of dosage and suggest that low-dose corticosteroids can achieve a good response in some circumstances, meriting validation in larger prospective clinical trials.

Our meta-analysis found the median duration of corticosteroid treatment to be 51.34 days and corticosteroid-only recovery time 47.59 days, suggesting that continual corticosteroid treatment after recovery is generally required in order to prevent relapse. These results should be interpreted with caution due to the small number of studies included in this analysis. Several studies mentioned a rebound in transaminases during corticosteroid tapering (27, 35, 36, 39, 44, 46, 50), and were generally consistent with the recommended guideline for prednisone tapering over 1 month. However, tapering over 1 year was also reported in an included study (44). Romanski et al. demonstrated no distinct association between recurrence of ILICI and steroid dose or reduction, although recurrence for those with high-grade ILICI and PD-1 inhibitors was more frequent (39). Reintroducing or increasing the dose of corticosteroids may be an effective measure in these cases (35, 46).

The greatest concern for prolonged use of corticosteroids or the use of high-dose corticosteroids is the effect on oncologic therapy and the emergence of adverse events, which partially restricts their use in clinical settings. One effect of high dose steroids is reduced immune function, increasing the risk for opportunistic infections (57). In one study, the majority of 31 patients treated with 20 mg or higher doses of prednisolone for at least 3 weeks experienced clinically significant side effects. Further, those who died from immunosuppressive therapy received more corticosteroids than those who did not (17), while spontaneous remission without corticosteroids improved prognosis. Even though some guidelines recommend initial observation without corticosteroid for low-grade ILICI, spontaneous improvement for severe ILICI was found in the included studies (34). However, grade 4 ILICI or acute liver failure requires immediate corticosteroid therapy (25). Further, steroids are known to inhibit antitumor responses in animal models. For clinical studies, results are mixed with high steroid doses negatively affecting ICI therapy, while other studies found the reverse (34, 48). A meta-analysis that evaluated the safety of corticosteroids and found no association between steroid use and ICI efficacy (56), thus quantitative synthesis with regard to efficacy is necessary to facilitate clinical decisions based on risk-benefit ratio.

Collectively, the dose and duration of steroid use need to be further optimized. Corticosteroid dose should be determined based on the patient’s pathological inflammatory status (58) and the duration of use should be built on the level of liver enzymes, comorbidities, the prospect of re-challenge with ICI, while minimizing the risk of adverse events. Further studies are needed to determine the efficacy of corticosteroids, the timing of steroid initiation, and the choice of second-line therapy. Decisions regarding corticosteroid therapy are currently based on clinical judgment and experience (48).

Of note, the ILICI corticosteroid response may be related to disease classification; hepatocellular, cholestatic, or mixed. However, a quantitative comparison of treatment effectiveness for these different types of liver injury was not conducted due to data limitations. Earlier studies have shown that the effectiveness of corticosteroids varies with distinct liver injury patterns, thus specific strategies need to be developed for each. In one study, corticosteroids produced an excellent response in most patients with hepatocellular liver injury, but fewer than 50% of patients with cholestatic disease improved with corticosteroids (25). Distinguishing between immune-mediated cholangitis (IMC) and immune-mediated hepatitis (IMH) is crucial for prediction of the response to corticosteroids. Cholestasis generally manifests as increased bile enzymes, such as elevated ALP, which may imply the development of IMC (28). In a systematic review, the effectiveness of corticosteroids used alone for the treatment of IMC was 11.5%, while the effectiveness of a combination of corticosteroids and UDCA was 28.6% (59). Thus, IMC didn’t respond well to corticosteroid therapy, but corticosteroids coupled with UDCA was a better choice in that an early application of corticosteroids controlled the inflammatory response caused by ICI. Further, long-term use of UDCA can promote the repair of the bile duct (60). Liver biopsy is valuable as a means by which to distinguish the cause of different types of liver injury associated with the use of corticosteroids. Clinically, those who are refractory to corticosteroid treatment or those with increased bilirubin, but no biliary blockage, are more likely to benefit from liver biopsies. However, liver biopsy during ILICI treatment is debatable because of the hazards involved.

Second-line immunosuppressive agents require further investigation in that ILICI recurrence during corticosteroid tapering and the prevalence of corticosteroid-refractory cases continue to be of clinical concern (39). ASCO and ESMO recommendations for second-line immunosuppressants are to be used if no improvement is observed within 3 days following initiation of corticosteroid therapy (52, 55). However, studies have reported that 14 (11-24) days of low-dose and 8 (4-14) days of high-dose methylprednisolone are appropriate before initiation of second-line immunosuppression (27). It has been suggested that a second immunosuppressive agent should only be considered for patients who have experienced failure of high-dose steroid therapy (34). There are no clear biomarkers that predict clinical requirement for second-line immunosuppressants in addition to steroid therapy for ILICI. Future studies should assess the criteria and predictive factors for the transition from steroids to second-line immunosuppression (43).

Second-line immunosuppressants are generally combined with steroids therapy for corticosteroid resistance. Although autoimmune hepatitis and ILICI are similar, the recommended treatment for the former is azathioprine with corticosteroids (61), while the latter is often steroids and MMF. MMF used for ILICI was found to have a high treatment response rate (93%) in this study. Luo et al. demonstrated steroid-refractory hepatitis to respond to MMF with good overall performance, which is consistent with our results (17). However, MMF and tacrolimus have potent anti-lymphocyte effects that impede lymphocyte-driven tumor surveillance, which may lead to rapid cancer progression. At this time, there is insufficient evidence to provide a clear recommendation for the most appropriate immunosuppressive therapy (62). It is worth noting that a prospective clinical trial exploring the most appropriate immunosuppressive regimen is currently underway (NCT04810156). Future studies should focus on treatment optimization and definition of treatment details.

The time to recover from treatment with immunosuppression (including corticosteroids) in our pooled analysis was 37.74 days. This period of time is shorter than corticosteroid therapy usage and is therefore consistent with previous studies that demonstrated early or concurrent second-line immunosuppressive therapy improved ALT in patients with grade 3 ILICI, reducing total steroid exposure (63). Another retrospective investigation also found that ALT declined more slowly in patients solely treated with steroids compared to those with a second immunosuppressant, suggesting that the addition of the second immunosuppressant accelerated the time to resolution without adversely compromising survival (43). Taken together, these findings indicate that the early use of second-line immunosuppressants is superior to long-term corticosteroid therapy.

These findings should be interpreted within the context of the inherent limitations to a meta-analysis that includes case series and retrospective studies, which introduce heterogeneity and complexity. First, compared to randomized controlled trials, case series and retrospective studies frequently provide a lower quality of evidence. However, prospective studies in this field are difficult to accomplish, hence current therapeutic strategies are primarily derived from clinical management. Second, several of the included studies failed to provide comprehensive immunosuppressant details, including dosage and duration of administration. Further, the response to immunosuppression for various patterns of liver injury was not well characterized, thus limiting quantitative subgroup analysis. Third, while clinical therapeutic management of ILICI adhered to guideline recommendations, physician’s judgment is also involved, which may influence immunosuppressive treatment outcomes. Finally, diagnosis and treatment of ILICI evolves with clinical practice and as a result treatment response rates and time to resolution can change over time.

## 5 Conclusion

For ILICI management, this study identified high response rates and good clinical effectiveness for two commonly used immunosuppressive agents, corticosteroids and MMF. Patient treatment with these two immunosuppressive agents is appropriate for most cases of ILICI. Future treatment approaches are likely to become more personalized, with the expectation that the response to immunosuppressive therapy will improve. This pooled analysis of median time to recovery for patients with ILICI and the duration of corticosteroid therapy is a beneficial guide for patient and physician expectations. Previously, a meta-analysis of the safety of corticosteroid therapy was completed and our work adds to the evidence supporting the effectiveness of immunosuppression, allowing for risk-benefit ratio considerations for treatment decisions and for optimal therapeutic choices. Further, this retrospective meta-analysis complements the ongoing prospective trial evaluating the effectiveness of immunosuppression for ILICI, within the context of standard clinical practice, providing for unique insight into future ILICI management

## Data availability statement

The original contributions presented in the study are included in the article/**Supplementary Material**. Further inquiries can be directed to the corresponding author.

## Author contributions

All authors contributed to the study conception and design. Literature Search, data collection and analysis were performed by KC, JH and JC. The first draft of the manuscript was written by KC, JX, JC and all authors commented on previous versions of the manuscript. All authors read and approved the final manuscript.
