# Supplementary material for: Effectiveness of immunosuppressant use for the treatment of immune checkpoint inhibitor-induced liver injury: A systematic review and meta-analysis
Source: Front Oncol. 2023 Mar 24;13:1088741. doi: 10.3389/fonc.2023.1088741 (PMC10080119; doi:10.3389/fonc.2023.1088741)
Supplement: Supplementary file 1 [file DataSheet_1.docx]

Supplementary Material

# Supplementary Data

**Supplementary 1:** Search strategy.

((((((((((((((Immune Checkpoint Inhibit*[Title/Abstract]) OR (Immune Checkpoint Block*[Title/Abstract])) OR (PD-1-PD-L1 Blockade[Title/Abstract])) OR (immunotherapy[Title/Abstract])) OR (PD-1 Inhibitor*[Title/Abstract])) OR (Programmed Cell Death Protein 1 Inhibitor*[Title/Abstract])) OR (PD-L1 Inhibitor*[Title/Abstract])) OR (Programmed Death-Ligand 1 Inhibitor*[Title/Abstract])) OR (CTLA-4 Inhibitor*[Title/Abstract])) OR (Cytotoxic T Lymphocyte Associated Protein 4 Inhibitor*[Title/Abstract])) OR (anti-PD1[Title/Abstract])) OR (anti-PDL1[Title/Abstract])) OR (Anti-CTLA-4[Title/Abstract])) OR (((((((((((((((((((((Nivolumab[MeSH Terms]) OR (Opdivo[Title/Abstract])) OR (Pembrolizumab[Supplementary Concept])) OR (Keytruda[Title/Abstract])) OR (Toripalimab[Supplementary Concept])) OR (Sintilimab[Supplementary Concept])) OR (Cemiplimab[Supplementary Concept])) OR (Libtayo[Title/Abstract])) OR (Tislelizumab[Supplementary Concept])) OR (Camrelizumab[Supplementary Concept])) OR (carrelizumab[Title/Abstract])) OR (Atezolizumab[Supplementary Concept])) OR (Tecentriq[Title/Abstract])) OR (Avelumab[Supplementary Concept])) OR (bavencio[Title/Abstract])) OR (Durvalumab[Supplementary Concept])) OR (Imfinzi[Title/Abstract])) OR (Ipilimumab[MeSH Terms])) OR (Yervoy[Title/Abstract])) OR (Tremelimumab[Supplementary Concept])) OR (ticilimumab[Title/Abstract]))) AND (((((((((((immune-related adverse event*[Title/Abstract]) OR (immune-related toxicit*[Title/Abstract])) OR (induced toxicit*[Title/Abstract])) OR (induced adverse event*[Title/Abstract])) OR (Liver Toxicit*[Title/Abstract])) OR (Liver Injur*[Title/Abstract])) OR (Hepatitis[Title/Abstract])) OR (Hepatitides[Title/Abstract])) OR (Liver Disease*[Title/Abstract])) OR (Hepatotoxicit*[Title/Abstract])) OR (Hepatic[Title/Abstract])).

# Supplementary Figures


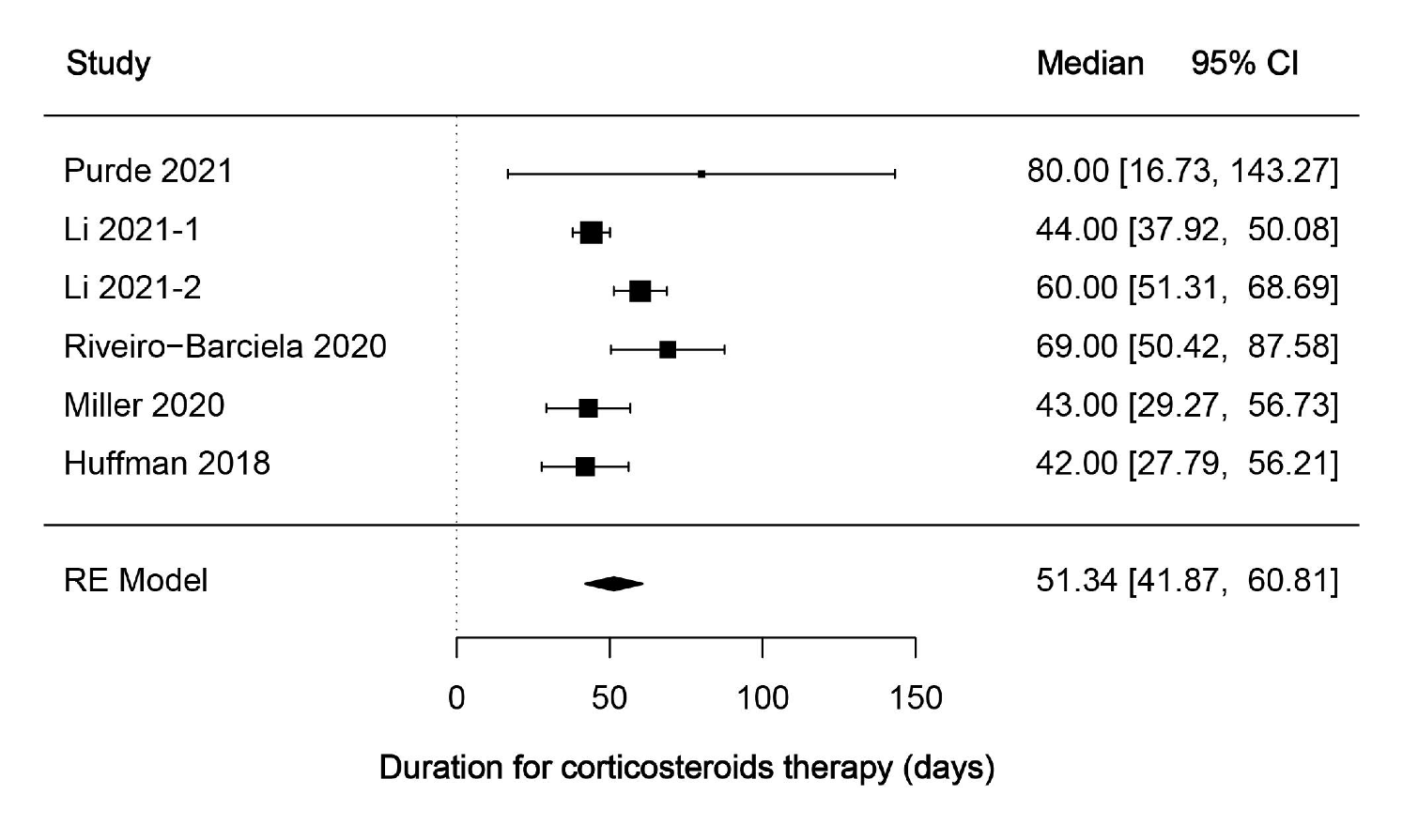


Supplementary Figure 1: Forest plots of duration for corticosteroids therapy.


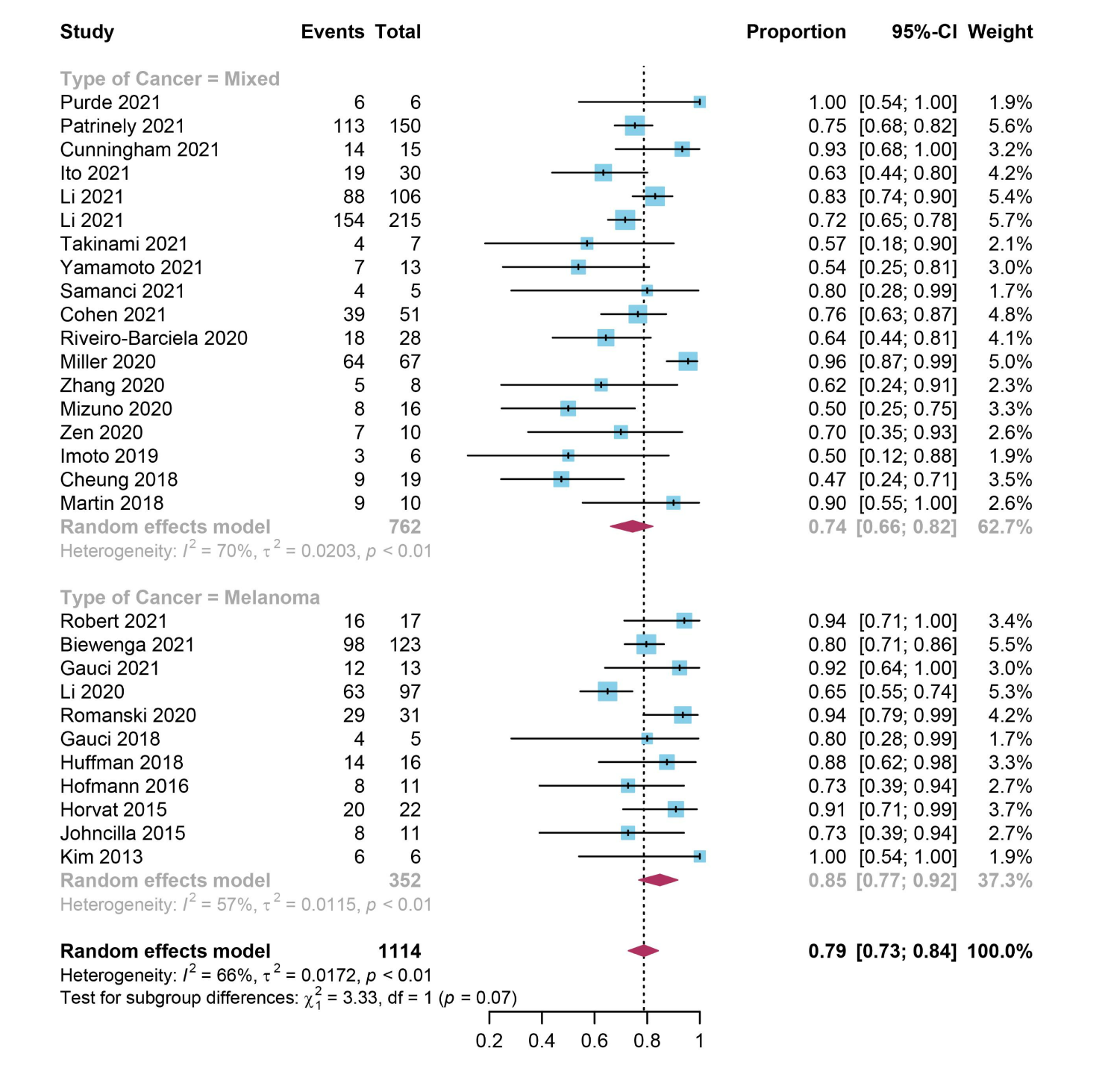


Supplementary Figure 2: Forest plots of pooled response rate to corticosteroids in patients with checkpoint inhibitor induced-liver injury according to type of cancer.


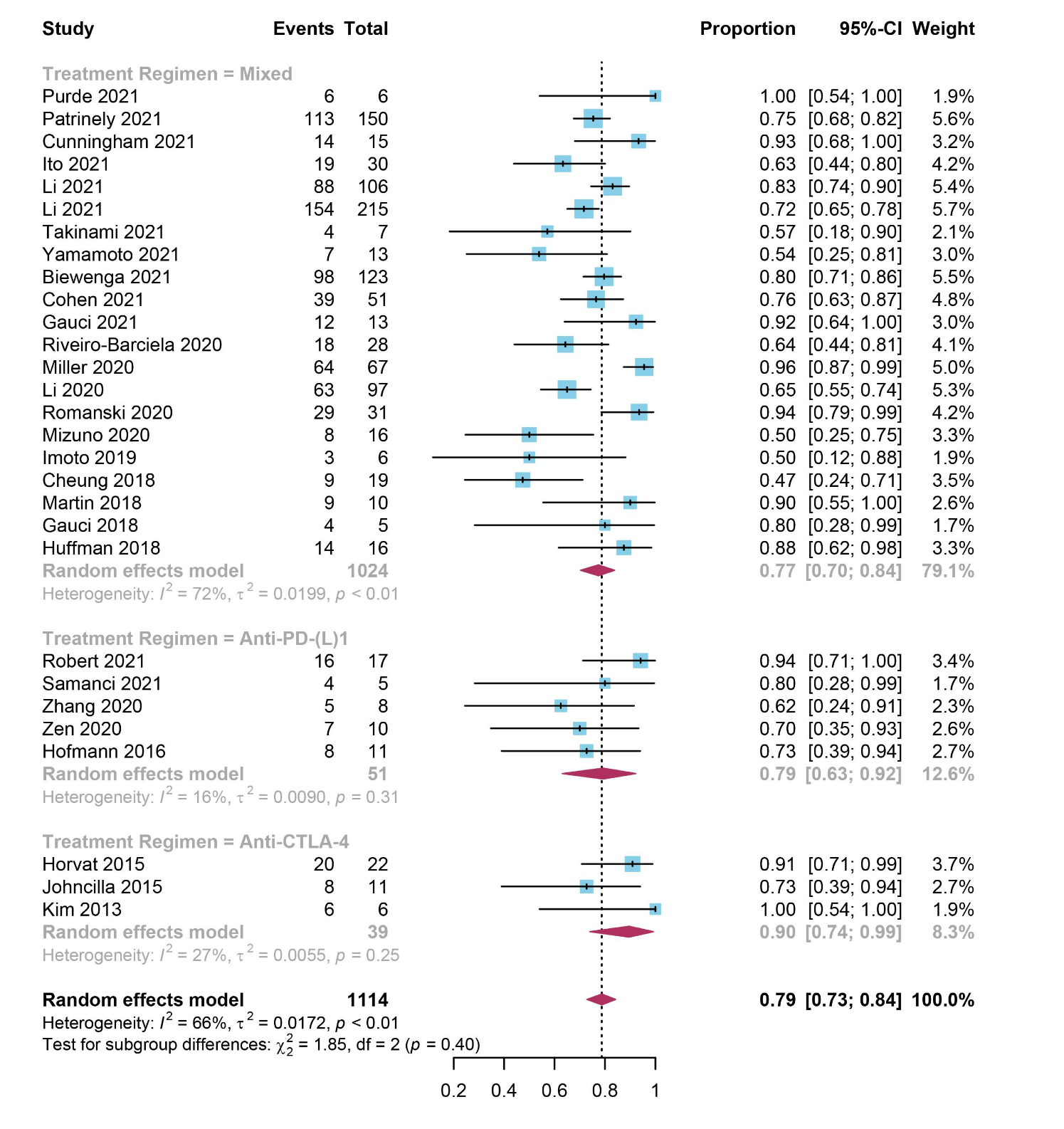


Supplementary Figure 3: Forest plots of pooled response rate to corticosteroids in patients with checkpoint inhibitor induced-liver injury according to treatment regimens.


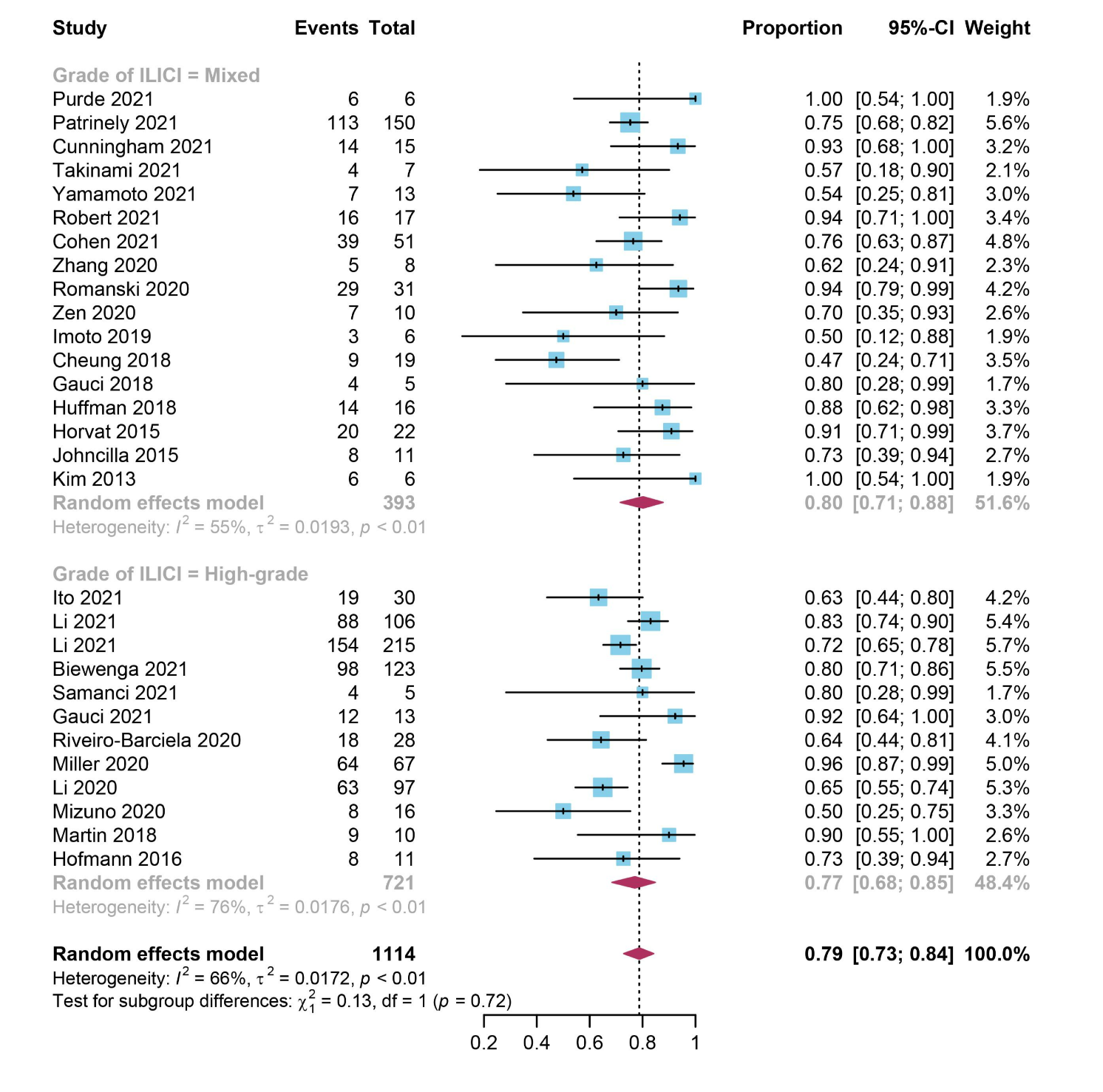


Supplementary Figure 4: Forest plots of pooled response rate to corticosteroids in patients with checkpoint inhibitor induced-liver injury according to grade of liver injury.


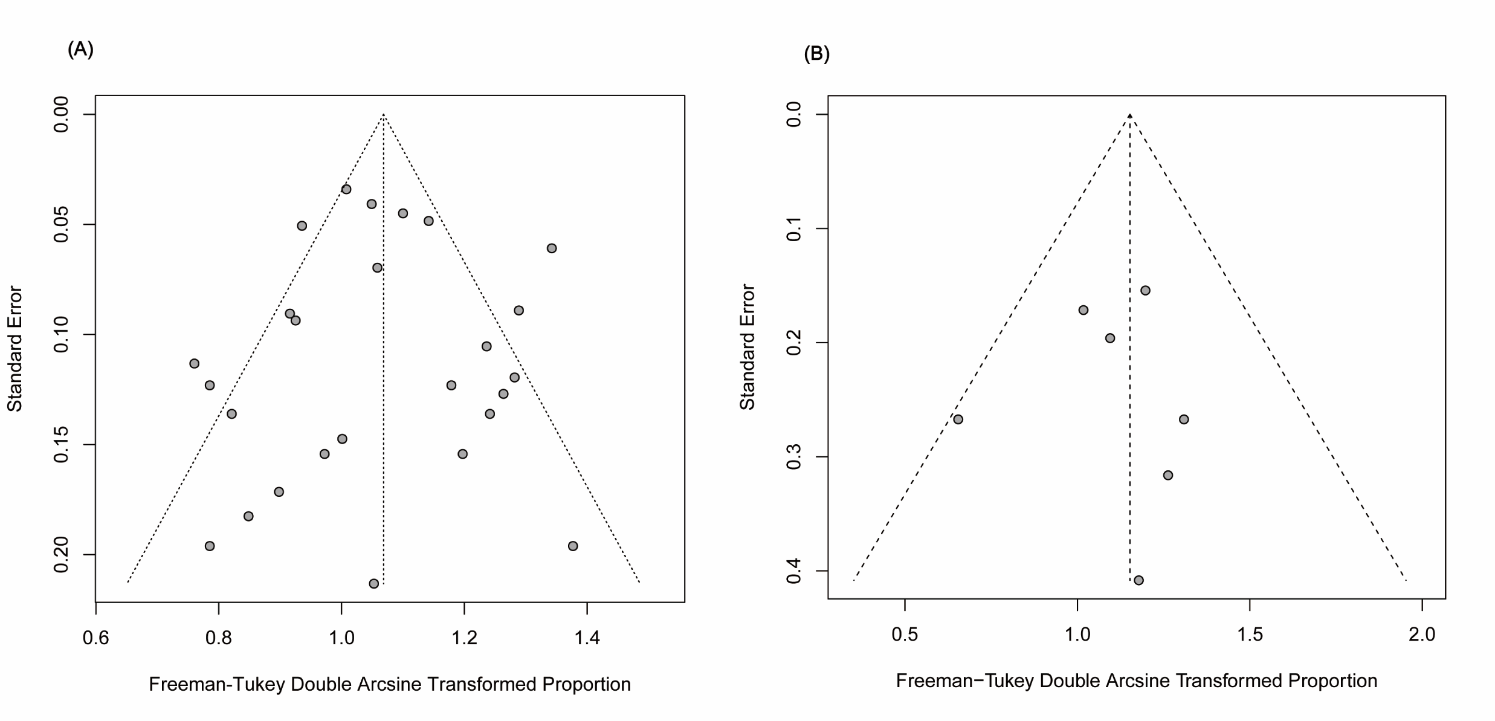


Supplementary Figure 5: (A) Funnel plots for efficacy of corticosteroids; (B) Funnel plots for efficacy of mycophenolate mofetil.

# Supplementary Tables

**Supplementary Table 1.** Risk of bias evaluated with the quality appraisal tool of Moga *et al*. which uses 18 criteria (“√” means criterion met; “×” means criterion not met).

| **Study** | **Study Objective** | | | | | | **Intervention and co-intervention** | | **Outcome measures** | | | **Statistical analysis** | **Results and conclusions** | | | | | **Competing interest and source of support** | **Total** |
| --- | --- | --- | --- | --- | --- | --- | --- | --- | --- | --- | --- | --- | --- | --- | --- | --- | --- | --- | --- |
|  | **1** | **2** | **3** | **4** | **5** | **6** | **7** | **8** | **9** | **10** | **11** | **12** | **13** | **14** | **15** | **16** | **17** | **18** |  |
| Purde *et al*, 2021 | √ | √ | × | √ | √ | √ | × | × | × | √ | √ | √ | √ | √ | × | × | √ | √ | 12 |
| Patrinely *et al*, 2021 | √ | √ | √ | √ | √ | × | √ | √ | √ | √ | √ | √ | √ | × | × | √ | √ | √ | 15 |
| Cunningham *et al*, 2021 | √ | √ | × | √ | √ | × | √ | √ | √ | √ | √ | √ | √ | × | × | √ | √ | √ | 14 |
| Ito *et al*, 2021 | √ | √ | √ | √ | √ | × | √ | √ | √ | √ | √ | √ | √ | × | × | × | √ | √ | 14 |
| Li *et al*, 2021 | √ | √ | × | √ | √ | × | √ | × | √ | √ | √ | √ | × | × | × | × | √ | √ | 11 |
| Li *et al*, 2021 | √ | √ | √ | √ | √ | × | √ | × | √ | √ | √ | √ | × | × | √ | √ | √ | √ | 14 |
| Takinami *et al*, 2021 | √ | √ | × | √ | √ | × | × | √ | √ | √ | √ | √ | × | × | × | × | √ | √ | 11 |
| Yamamoto *et al*, 2021 | √ | √ | √ | √ | √ | × | √ | √ | √ | √ | √ | √ | √ | × | × | × | √ | √ | 14 |
| Robert *et al*, 2021 | √ | √ | √ | × | √ | × | × | × | × | √ | √ | √ | √ | × | √ | × | √ | √ | 11 |
| Luo *et al*, 2021 | √ | √ | × | √ | √ | × | √ | × | √ | √ | √ | √ | √ | √ | × | √ | √ | √ | 14 |
| Biewenga *et al*, 2021 | √ | √ | √ | √ | √ | × | × | × | × | √ | √ | √ | √ | × | × | × | √ | √ | 11 |
| Samanci *et al*, 2021 | √ | √ | × | √ | √ | × | √ | √ | × | √ | √ | √ | × | × | × | × | √ | √ | 11 |
| Cohen *et al*, 2021 | √ | √ | × | √ | × | × | × | × | √ | √ | √ | × | × | × | × | × | √ | √ | 8 |
| Gauci e*t al*, 2021 | √ | √ | × | √ | √ | × | √ | × | √ | √ | √ | √ | × | × | √ | × | √ | √ | 12 |
| Riveiro-Barciela *et al*, 2020 | √ | √ | × | √ | √ | × | √ | √ | × | √ | √ | √ | × | × | × | √ | √ | √ | 12 |
| Miller *et al,* 2020 | √ | √ | × | √ | √ | × | × | √ | √ | √ | √ | √ | √ | × | × | × | √ | √ | 12 |
| Li *et al*, 2020 | √ | √ | √ | √ | √ | × | √ | √ | × | √ | √ | √ | √ | × | × | × | √ | √ | 13 |
| Zhang *et al*, 2020 | √ | √ | √ | × | × | × | × | √ | × | √ | √ | × | √ | × | × | × | √ | √ | 9 |
| Romanski *et al*, 2020 | √ | √ | × | √ | √ | × | √ | √ | √ | √ | √ | √ | × | × | × | × | √ | × | 11 |
| Mizuno *et al*, 2020 | √ | √ | √ | √ | √ | × | × | √ | × | √ | √ | √ | √ | × | × | × | √ | √ | 12 |
| Zen *et al*, 2020 | √ | √ | √ | × | × | × | √ | √ | √ | √ | √ | √ | × | × | × | × | √ | × | 10 |
| Imoto *et al*, 2019 | √ | √ | × | √ | √ | × | √ | √ | × | √ | √ | √ | × | × | × | × | √ | √ | 11 |
| Cheung *et al*, 2018 | √ | √ | × | √ | √ | × | √ | √ | √ | √ | √ | √ | √ | × | × | × | √ | √ | 13 |
| Martin *et al*, 2018 | √ | √ | × | √ | √ | × | √ | √ | √ | √ | √ | √ | × | × | × | × | √ | √ | 12 |
| Gauci *et al*, 2018 | × | √ | × | × | √ | × | √ | √ | × | √ | √ | × | × | × | × | × | √ | √ | 8 |
| Huffman *et al*, 2018 | √ | √ | × | √ | √ | × | √ | √ | √ | √ | √ | √ | × | × | × | × | √ | √ | 12 |
| Hofmann *et al*, 2016 | √ | √ | √ | × | √ | √ | √ | √ | × | √ | √ | × | × | × | × | × | √ | √ | 11 |
| Horvat *et al*, 2015 | √ | √ | × | √ | √ | × | × | √ | × | √ | √ | √ | × | × | × | × | √ | √ | 10 |
| Johncilla *et al*, 2015 | √ | √ | √ | √ | × | × | √ | √ | × | √ | √ | × | × | × | × | × | √ | √ | 10 |
| Kim et al, 2013 | √ | √ | × | √ | × | × | √ | × | × | √ | √ | × | × | × | × | × | √ | √ | 8 |

**Note:**

**Study Objective** 1. Is the hypothesis/aim/objective of the study clearly stated in the abstract, introduction or methods section? Study population 2. Are the characteristics of the participants included in the study described? 3. Were the cases collected in more than one center? 4. Are the eligibility criteria (inclusion and exclusion criteria) to enter the study explicit and appropriate? 5. Were participants recruited consecutively? 6. Did participants enter the study at a similar point in the disease?

**Intervention and co-intervention** 7. Was the intervention clearly described in the study? 8. Were additional interventions (co-interventions) clearly reported in the study?

**Outcome measures** 9. Are the outcome measures clearly defined in the introduction or methods section? 10. Were relevant outcomes appropriately measured with objective and/or subjective methods? 11. Were outcomes measured before and after intervention? Statistical analysis 12. Were the statistical tests used to assess the relevant outcomes appropriate? Results and conclusions 13. Was the length of follow-up reported? 14. Was the loss to follow-up reported? 15. Does the study provide estimates of the random variability in the data analysis of relevant outcomes? 16. Are adverse events reported? 17. Are the conclusions of the study supported by results?

**Competing interest and source of support** 18. Are both competing interest and source of support for the study reported?
